# Characterization of Pedestrian Crossing Spatial Violations and Safety Impact Analysis in Advance Right-Turn Lane

**DOI:** 10.3390/ijerph19159134

**Published:** 2022-07-26

**Authors:** Ziyu Chen, Xiufeng Chen, Ruicong Wang, Mengyuan Gao

**Affiliations:** School of Mechanical and Automotive Engineering, Qingdao University of Technology, Qingdao 266520, China; chenziyu1997@yeah.net (Z.C.); 18832055455@163.com (R.W.); gaomengyuan4599@163.com (M.G.)

**Keywords:** traffic safety, advance right-turn lane, spatial crossing violation, conflict severity, multivariate ordered logistic model

## Abstract

In view of the pedestrian space violation in an advance right-turn lane, the pedestrian crossing paths are divided by collecting the temporal and spatial information of pedestrians and motor vehicles, and the characteristics of different pedestrian crossing behaviors are studied. Combined with the time and speed indicators of conflict severity, the K-means method is used to divide the level of conflict severity. A multivariate ordered logistic regression model of the severity of pedestrian–vehicle conflict was constructed to quantify the effects of different factors on the severity of the pedestrian–vehicle conflict. The study of 1388 pedestrians and the resulting pedestrian–vehicle conflicts found that the type of spatial violation has a significant impact on pedestrian crossing behavior and safety. The average crossing speed and acceleration variation values of spatially violated pedestrians were significantly higher than those of other pedestrians; there is a significant increase in the severity of pedestrian–vehicle conflicts in areas close to the oncoming traffic; the average percentage of pedestrian–vehicle conflicts due to spatial violations increased by 12%, and the percentage of serious conflicts due to each type of spatial violation increased from 18% to 87%, 74%, 30%, and 63%, respectively, compared with those of non-violated pedestrians. In addition, the decrease in the number of lanes and the increase in speed and vehicle reach all lead to an increase in the severity of pedestrian–vehicle conflicts. The results of the study will help traffic authorities to take measures to ensure pedestrian crossing safety.

## 1. Introduction

Urban development brings a rapid growth in traffic volume, and a series of problems such as traffic congestion and traffic safety, which are becoming more and more serious. Traffic safety has always been a hot issue in the field of traffic engineering. In recent years, approximately 200,000 traffic accidents have occurred in China [1]. As a vulnerable group of road users, pedestrians are more likely to be injured in traffic accidents. According to statistics, nearly a quarter (23%) of the 1.35 million deaths due to road traffic collisions worldwide each year are pedestrians, with urban roadway casualties accounting for approximately 73% of the total [2,3]. This is mainly due to the rapid population growth and the increase in the number of motor vehicles in the city center, as well as non-compliance with traffic rules, and other dangerous behaviors by pedestrians, drivers, and other road users [4,5]. Therefore, the study of pedestrian crossing behavior and interaction with vehicles is important for traffic safety.

Currently, advance right-turn lanes are a common design option in China at intersections with high right-turn traffic volumes. This form consists of a channelized island and a right-turn lane, in order to improve the efficiency of right-turning vehicles [6]. This design splits the right-turn traffic flow in advance by setting the right-turn lane, but this channelization method improves the efficiency of the right-turn vehicle. At the same time, because pedestrians and motor vehicles are not constrained by signal control, it increases the risk of conflict or even of traffic accidents between pedestrians and motor vehicles. In addition to the impact of the absence of signal control, different behaviors of pedestrians can directly affect their safety. Additionally, these behaviors are closely related to their age, gender, road environment, and other factors [7,8,9]. Therefore, it is of great significance to study the behavioral characteristics of pedestrians at the advance right-turn lanes and the safety issues.

Most studies on pedestrian–vehicle conflicts focus on pedestrians who do not obey traffic rules and who run red lights illegally, causing pedestrian–vehicle conflicts. Additionally, most of the selected factors regarding pedestrians’ personal factors are physiological differences, cell phone influence, etc. However, in the operation of urban traffic, the problem of pedestrian–vehicle conflicts without signal control is more common, especially in the advance right-turn lane. Some pedestrians use crosswalks irregularly to cross the street because they want a closer crossing path, which has significant impact on pedestrian–vehicle conflicts, but is often ignored by scholars. Therefore, this paper combines the research of other scholars to classify the pedestrian use of crosswalks as one of the factors in the analysis of pedestrian–vehicle conflicts. Based on the video data collected in the field, pedestrian characteristics are recorded, and pedestrian–vehicle trajectory data are extracted. The K-means method was used to classify the severity levels of pedestrian–vehicle conflicts, and a multivariate ordered logistic regression model was established to quantify the effects of different factors on the severity of pedestrian–vehicle conflicts.

## 2. Literature Review

With the development of computer vision technology, the number and accuracy of traffic conflict samples has also been improved. Scholarly research on pedestrian crossing safety has expanded from a single perspective of pedestrians or motor vehicles to a multi-angled, comprehensive analysis of people, vehicles, roads, and environments. In terms of model selection, the logistic model is widely used in the field of traffic safety, because it is suitable for multi-factor analysis.

### 2.1. Pedestrian Crossing

The pedestrian crossing problem has been extensively studied worldwide, focusing on two scenarios: signal control and non-signal control. Aghabayk et al. analyzed the characteristics of pedestrian crossing behavior at unsignalized intersections by considering pedestrian characteristics, environment, and technology as influencing factors, and they developed a linear model to analyze the variability of each factor [9]. Mwakalonge et al. studied the effect of distraction on pedestrian crossing behavior. The authors found that 29.8% of the 1102 pedestrians showed distraction activities when crossing the road, such as eating snacks, using handheld devices, reading, and listening to music. These activities led to significant differences in pedestrian crossing behaviors [10]. Hediyeh et al. conducted a study on pedestrian differences and analyzed the effects of gender, age, the number of pedestrians in the population, crosswalk length, and signal phase on pedestrian movement status, to understand the impact of intersection characteristics on the pedestrian crossing at a micro level [11]. For the selection of pedestrian crossing behavior indicators, most scholars choose walking speed [12,13,14], the number of stops [15], and whether there is observation behavior [16,17] for analysis. However, the irregular use of pedestrian crosswalks by pedestrians has not been given too much attention, and not many studies have been conducted on it. In practice, however, this behavior is very common and has a significant impact on human–vehicle interaction, and should therefore be taken seriously.

### 2.2. Pedestrian–Vehicle Conflict

A traffic accident is a small probability event, and most scholars take traffic conflict as an alternative evaluation index in the interaction between pedestrians and motor vehicles. Yuan Li et al. introduced the idea of physical dynamics and took the crosswalk of the non-signal control section as the research object. Considering the probability of pedestrian–vehicle conflict, the severity of the conflict, and the expected conflict of pedestrian vehicles, the risk assessment model of pedestrian–vehicle conflict was constructed [18]. Zhang employed a game-theory approach to investigate the joint behaviors of pedestrians and motorists from the perspective of safety [19]. Muley et al. demonstrated the use of a microsimulation environment to predict vehicle–vehicle and pedestrian–vehicle conflicts at signalized intersections [20]. Peng Yong et al. studied the method of classifying pedestrian–vehicle conflict levels on unsignalized controlled road sections, and selected 13 independent explanatory variables in three aspects: people, vehicles, and roads, to establish a quantitative model of pedestrian–vehicle conflict based on the Ordered Probit model, and conducted significant analysis of the effect of variables on pedestrian–vehicle conflict levels [21]. Zhang et al. analyzed the conflict process between right-turning motor vehicles and pedestrians, clarified right-of-way in the conflict as well as the operation mechanism, and quantified the conflict impact based on the microscopic characteristics of pedestrian–motor vehicle conflict, with delay as an indicator [22]. However, in the analysis of the severity of pedestrian–vehicle conflicts, most studies only classify conflicts from a single indicator, which cannot accurately or better describe the severity of conflicts.

### 2.3. Advance Right-Turn Lane

Advance right-turn lanes are widely implemented to improve the safety and mobility of traffic. Jiang et al. investigated the impact of channelized right turns on pedestrian safety, based on surrogate safety and behavior measures [23]. Chen et al. used the ratio method to quantitatively analyze the severity of the conflict between pedestrians and vehicles in the early right-turn lane, and obtained the influence law of factors such as pedestrian crossing behavior, speed, and crossing position on the severity of the conflict [24].

Although some studies have conducted studies of pedestrian–vehicle conflicts at advance right-turn lanes, they have not linked individual pedestrian differences and dangerous crossing behaviors to pedestrian–vehicle conflicts. Most studies have ignored the impact of pedestrian space violations on traffic safety. Therefore, in this paper, the study of pedestrian–vehicle safety at the advance right-turn lane will be combined with pedestrian crossing trajectories to explore the effects of different factors on pedestrian–vehicle conflicts.

## 3. Data Description

### 3.1. Pedestrian Crossing Path Classification

**Figure 1:**
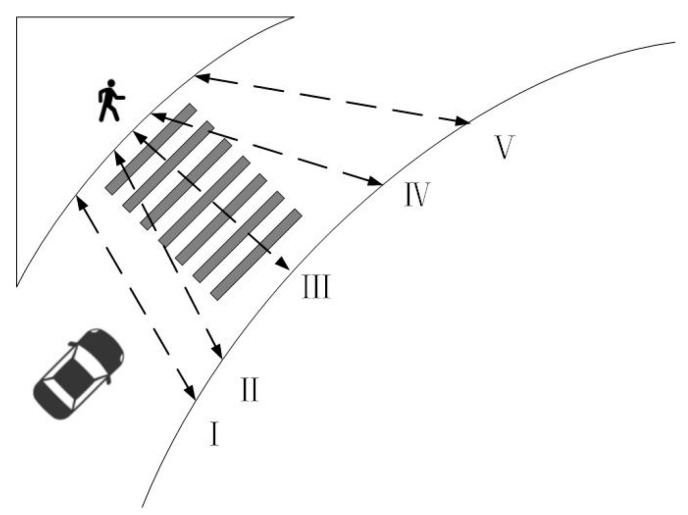
Pedestrian crossing path type.

Pedestrian crossing behavior is affected by the surrounding environmental factors. Large shopping malls, transportation hubs, and other attractive facilities will lead to pedestrians choosing the shortest path to reach the destination, due to their psychological proximity, resulting in path deviation from the pedestrian crosswalk [25]. At the advance right-turn lane, the pedestrian crossing paths are divided into five types according to the location distribution of pedestrians deviating from the crosswalk, as shown in Figure 1. Pedestrian paths I and V are for pedestrians crossing the street without using the crosswalk at all. Pedestrian paths II and IV are for pedestrians who partially use the crosswalk to cross the street, and path III is for pedestrians who fully use the crosswalk to cross the street. Except for pedestrian path type III, all other crossing types are pedestrian spatial violations [26].

### 3.2. Data Collection and Processing

#### 3.2.1. Data Collection

**Figure 2:**
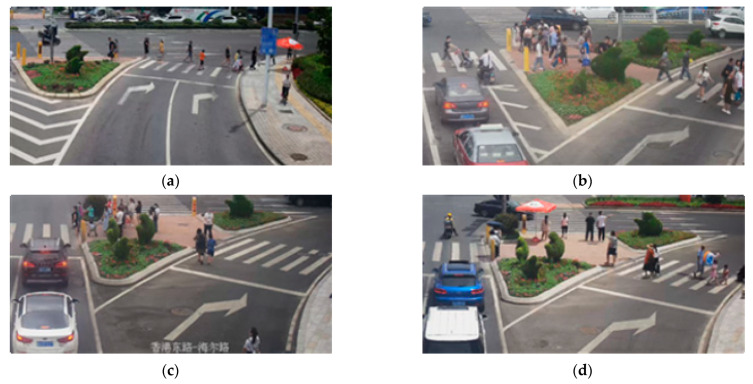
Investigation Site: (**a**) Site 1; (**b**) Site 2; (**c**) Site 3; (**d**) Site 4.

This study selected four advance right-turn lanes at the intersection of Hong Kong Middle Road-Haier Road in Qingdao as the investigation sites (Figure 2). The intersections are surrounded by attractive larger buildings (shopping malls, subway stations, etc.), which effectively reduce the influence of environmental differences on pedestrian crossing paths.

The survey time is the peak time of population flow from 18:00 to 19:00 on holidays, and the specific information of the survey site is shown in Table 1. In this paper, a total of 1388 pedestrian samples were observed, of which 42.58% of the total number of pedestrians crossed the street with spatial violations. Among the different spatial violation path types, the highest percentage of pedestrians was found in type IV paths (34.86%), followed by type II paths (33.00%).

#### 3.2.2. Data Processing

**Figure 3:**
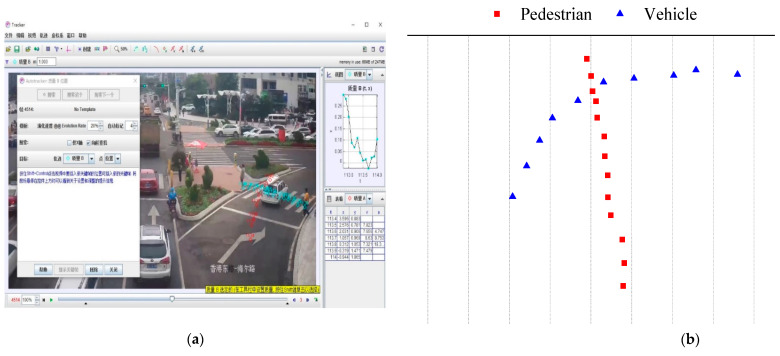
Track extraction transformation: (**a**) Software Screenshot; (**b**) Extraction coordinates.

The experimenter uses Tracker to process the captured video, and extracts pedestrian and vehicle path information using the tracked target trajectory function in Tracker, as shown in Figure 3a. The trajectory is then converted to coordinate information, as in Figure 3b. Researchers judge and record pedestrian physiological information such as gender, age, etc. The survey data include gender, age, the impact of technological equipment, conflict with vehicles, pedestrian crossing speeds, vehicle speeds, vehicle arrival rates, and pedestrian arrival rates, as shown in Table 2. Pedestrian individual differences were recorded, and we adopted a group assignment method to improve the accuracy of the experimentalists’ observation records [27]. For the recording of age, we divided age into 5 groups according to size: children (<10), teenagers (10–20), youth (20–40), middle-aged (40–60), and elderly (>60). Regarding the recording of velocity, this paper uses the extracted trajectory coordinates for calculation. We use a portion of the sample to train the experimenters before processing the data to minimize the effect of errors from observations.

## 4. Methodology

### 4.1. Study on Pedestrian Crossing Behavior

This paper adopts the method of cross analysis when studying pedestrian behavior. The types of pedestrian crossing paths previously classified as behavioral indicators are used to study the differences in crossing paths by different factors. The effects of different crossing paths on pedestrian speed and the probability of pedestrian–vehicle conflict are compared horizontally.

### 4.2. Study on Pedestrian–Vehicle Conflict

#### 4.2.1. Pedestrian–Vehicle Conflict Indicator

This paper mainly studies the influence of factors on the severity of the pedestrian–vehicle conflict. Different from the conflict between motor vehicles, when the conflict occurs between pedestrians and motor vehicles, due to the complex changes in motion state, a single indicator cannot better describe the severity of the conflict. Therefore, this paper selects three types of indicators: Time to Conflict (TTC) [28], Post-Encroachment Time (PET) [29], and Deceleration-to-Safety (DST) [30,31] as the discriminant indicators of conflict severity. TTC means that the vehicle keeps its current state of motion unchanged until the collision occurs; the smaller the TTC value, the greater the likelihood of an accident. DST is the maximum acceleration for traffic participants to change their movement to avoid conflict; the larger the DST value, the more serious the conflict occurring. PET is the time difference between the former leaving the conflict zone and the latter reaching the conflict zone in traffic conflicts [28]. According to the collected coordinate information, the human–vehicle conflict severity index is calculated by homogeneous coordinate transformation, as shown in Formulas (1)–(3). After collecting the pedestrian–vehicle conflict indicators, the K-means method is used to cluster the conflicts and to classify the severity levels of pedestrian–vehicle conflicts for modeling and analysis.

(1) $$\mathrm{TTC}=\min\{\max(\frac{S_{ci}}{V_{ci}},\frac{S_{pi}}{V_{pi}})\}$$

(2) $$\mathrm{DST}=\max\{\left|\frac{2V_{pi}(S_{pi}V_{ci}-S_{ci}V_{pi})}{S_{pi}^{2}}\right|\}$$

(3) $$\mathrm{PET}=T_{l}-T_{a}$$

where: $T_{l}$ is the moment when the former leaves the conflict area, $T_{a}$ is the moment when the latter arrives at the conflict area, $S_{ci}$ is the distance of the motor vehicle from the conflict point (m), $S_{pi}$ is the distance of pedestrians from the conflict point (m), $V_{ci}$ is the speed of the motor vehicles (m/s), $V_{pi}$ is the speed of the motor vehicles and pedestrians (m/s).

#### 4.2.2. Pedestrian–Vehicle Conflict Severity Model

To explore the effects of different factors on the severity of the pedestrian–vehicle conflict, after completing the classification of the severity level of pedestrian–vehicle conflict, gender (*X*_1_), age group (*X*_2_), technological device (*X*_3_), type of crossing path (*X*_4_), vehicle speed (*X*_5_), vehicle arrival rate (*X*_6_), the number of lanes (*X*_7_), and pedestrian arrival rate (*X*_8_) were selected as independent variables, and the severity level of conflict as dependent variables. Firstly, the correlation analysis of the independent variables in the model is carried out to ensure that they meet the collinearity test. In this paper, SPSS software (IBM, Armonk, NY, USA) is used to select the Spearman coefficient for the correlation analysis of variables. The multivariate ordered Logistic model is often used in studies where the dependent variable is of multiple types, and there is an order or rank. The model can visualize the degree of influence of different factors on the dependent variable. Since transportation systems are multi-factor complex systems, this model is often used in the study of traffic safety. After completing the collinearity test, because the severity index of pedestrian–vehicle conflict has multiple levels, the more serious the conflict is, the more likely it is to meet the requirements of the multivariate ordered Logistic model for dependent variables. Therefore, a conflict severity model based on multivariate ordered Logistic model is established. The specific expression of the model is as follows:

(4) $$P=\frac{e^{\left(\alpha +\beta_{1}X_{1}+\beta_{2}X_{2}+...+\beta_{8}X_{8}\right)}}{1+e^{\left(\alpha +\beta_{1}X_{1}+\beta_{2}X_{2}+...+\beta_{8}X_{8}\right)}}$$

Taking Logit change for *P*, the model is linearized as:

(5) $$LogitP=\ln(\frac{P_{i}}{1-P_{i}})=\alpha +\beta_{1}X_{1}+\beta_{2}X_{2}+...+\beta_{8}X_{8}$$

where: *P_i_* is the probability of severity level *i* conflict, *X*_1_~*X*_8_ is the independent variable, and *β*_1_~*β*_8_ is the corresponding coefficient, *α* is the constant term.

## 5. Result

### 5.1. Analysis of Pedestrian Crossing Behavior

#### 5.1.1. Characterization of Pedestrian Crossing Paths

**Figure 4:**
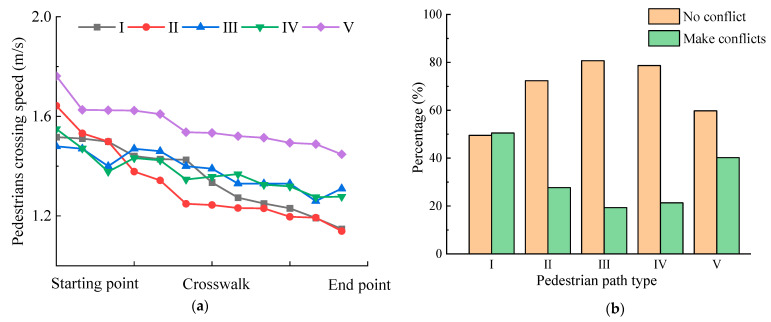
Crossing path impact: (**a**) Pedestrian crossing speed; (**b**) pedestrian–vehicle conflict.

Different types of pedestrian paths show different characteristics in crossing speed and pedestrian–vehicle conflict, as shown in Figure 4. It was observed that the average speed of pedestrians crossing the street is 1–1.8 m/s, and the speed is a decreasing trend, as shown in Figure 4a. The pedestrian crossing speed of path type V is significantly higher than that of other types, and the speed of pedestrians (type I and type II) near the direction of motor vehicles decreases by more than 20% before and after crossing. It can be seen from Figure 4b that spatial violations (except path type III) lead to an increase in the proportion of conflicts, and the proportion of pedestrian–vehicle conflicts in the path types I and V is as high as 50.49% and 40.23%, respectively.

#### 5.1.2. Analysis of Influencing Factors of Pedestrian Behavior

**Figure 5:**
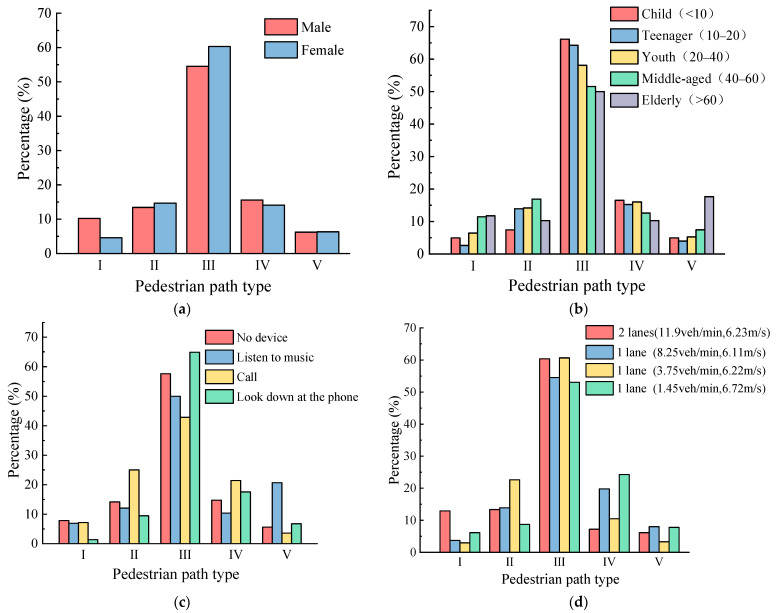
Cross analysis of crossing path types: (**a**) Gender; (**b**) Age; (**c**) Technological device; (**d**) Traffic conditions.

Different gender, ages, technology devices, vehicle arrival rates, vehicle speeds, and lane numbers have a significant influence on pedestrian crossing paths, as shown in Figure 5. Compared with female pedestrians (39.71%), male pedestrians’ spatial violations are more frequent (45.56%); especially for path type I, the percentage of male violations is significantly higher than that of females (Figure 5a), which shows that males are less aware of traffic safety. Figure 5b shows that an increase in age leads to an increase in irregular pedestrian use of crosswalks, especially for the elderly; the proportion of the elderly who do not use crosswalks (path type V) reaches 29.40%. As can be seen from Figure 5c, the degrees of influence of technological devices on pedestrian crossing space violations in descending order are: call (57.15%), listening to music (50%), no device (42.35%), and looking down at the phone (35.14%). The reason for this trend is that most of the pedestrians who look down at their phone actively observe the traffic flow and their position before crossing the street, and choose to cross the road inside of the crosswalk. Therefore, there are fewer spatial violations for pedestrians looking down at their phones. By comparing the four observation sites, it is clear that pedestrian spatial violations are significantly worse at locations with low vehicle arrival rates (Figure 5d). This conclusion is consistent with the results of Zhang’s theoretical research on the acceptable gap between pedestrians. The lower vehicle reaching rate corresponds to the larger vehicle gap, and pedestrians are more inclined to cross the road [32].

### 5.2. Pedestrian–Vehicle Conflict Analysis

#### 5.2.1. Conflict Severity Grading

**Figure 6:**
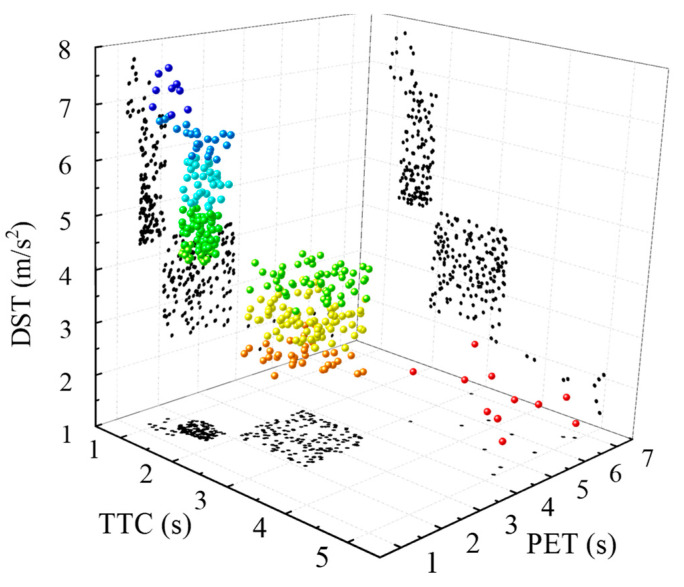
Conflict severity indicators.

According to Formulas (1)–(3) in Section 4, the 319 personal vehicle conflict samples collected in the experiment are calculated, and the scatter plot of conflict indicators is shown in Figure 6. The conflict is more serious, and some indicators are evenly distributed in the interval. The reason for this is that pedestrians often cross the street in groups. When they conflict with motor vehicles, the movement state of individuals in the crowd is affected by peers, resulting in similar or identical indicators of the severity of the pedestrian–vehicle conflict. From the results in the figure, it is obvious that the conflict indicator sizes show a clear aggregation feature, which is good for providing a reference for using clustering methods.

**Figure 7:**
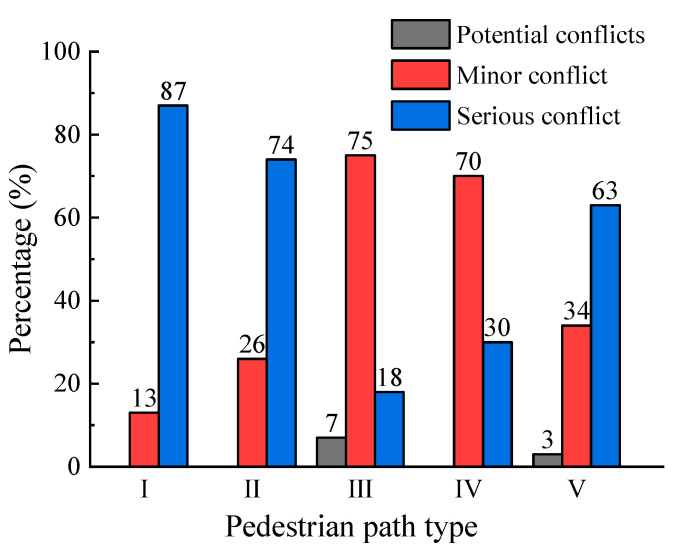
Percentage of conflict severity by path type.

Before clustering, the number of categories are first determined. Based on the results in Figure 6 and related studies [33,34,35], the number of categories k = 3 was determined as potential conflict, minor conflict, and serious conflict. The clustering results are obtained by the K-means method, and are shown in Table 3. Additionally, the reliability of each indicator in conflict severity classification is proven using one-way ANOVA analysis. The pedestrian–vehicle conflicts are classified according to the clustering results, and the conflict proportions of different severity under different pedestrian path types are obtained, as shown in Figure 7. The figure clearly shows that pedestrian path type III, i.e., pedestrians using the crosswalk in a regulated way, have the smallest proportion of serious conflicts occurring. For other pedestrian path types, the proportion of serious conflicts increased to different degrees, which proves that spatial violations have a significant effect on the severity of pedestrian–vehicle conflicts.

#### 5.2.2. Correlation Analysis of Independent Variables

**Figure 8:**
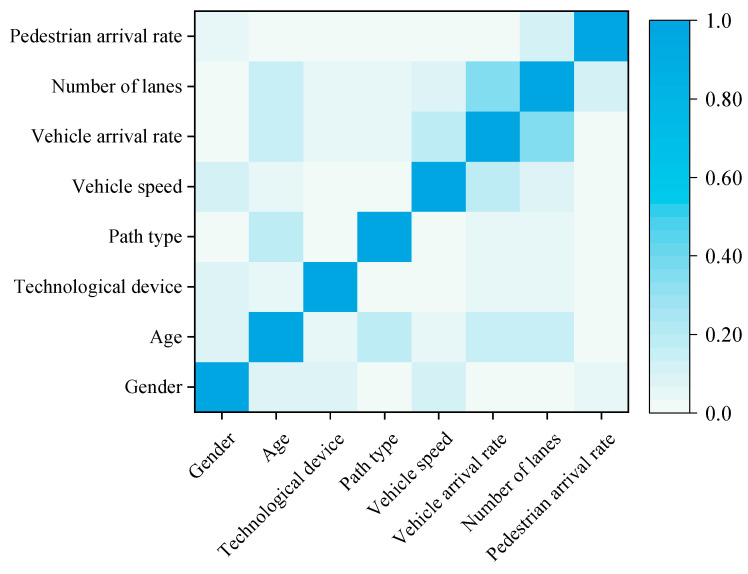
Correlation coefficient of independent variables.

Before conducting the multivariate ordered logistic regression analysis, the eight independent variables selected for this paper were first tested for correlation. The correlation between variables showed that the correlation coefficients were less than 0.4, and most of them were less than 0.1. There is no significant correlation between the variables, which meets the collinearity test and can be used for regression analysis (Figure 8).

#### 5.2.3. Analysis of Model Results

The model-dependent variable conflict severity is assigned according to the clustering results, with conflict severity = 1 (potential conflict), conflict severity = 2 (minor conflict), and conflict severity = 3 (serious conflict). The regression analysis results of pedestrian–vehicle conflict samples are shown in Table 4, and the reference dependent variable is conflict severity = 3 (serious conflict).

As shown in Table 4, for the traffic conditions, an increase in vehicle speed (4.495) and vehicle arrival rate (0.451) both increase the severity of pedestrian–vehicle conflicts. Motor vehicles approaching the right turn lane at higher initial speeds require higher braking speeds to avoid collisions when encountering pedestrians, thus increasing the severity of the resulting pedestrian–vehicle conflicts. In contrast, the effect of the pedestrian arrival rate on conflict severity is not significant. Compared to the elderly (>60), teenagers (−4.069) and the middle-aged (−1.666) had lower levels of conflict severity. The elderly are at the greatest risk of exposure to serious conflicts, due to their weak awareness of traffic safety. The conflict between motor vehicles and the elderly is more serious. The effect of different types of cell phone usage on conflict severity was not significant in the analysis of the impact of technological devices, with significance p-values of greater than 0.05. In the spatial violation analysis, the non-violation of pedestrians (path type III) greatly reduces the severity level of pedestrian–vehicle conflict (B = −2.527) compared to path type V. All spatial violations by pedestrians near the oncoming side of the vehicle resulted in an increase in the severity of pedestrian–vehicle conflict (path type I: B = 1.432; path type II: B = 0.636). In addition, the severity of conflict is inversely proportional to the number of lanes (B = 3.940), probably because pedestrians often choose to risk crossing the street due to the relatively short distance of crosswalks on roads with fewer lanes. Pedestrians often choose to accelerate the risk of crossing the street, resulting in a shorter reaction time between pedestrians and drivers, resulting in pedestrian–vehicle conflicts, and the severity of the conflict is at a high level.

## 6. Discussion 

The analysis of pedestrian–vehicle conflict in this paper innovatively incorporates the factor of pedestrian crossing paths and explores the impact of pedestrian spatial violations on traffic safety. However, there are still many improvements to be made. First, the study should consider more scenarios of pedestrian–vehicle conflicts, such as the effects of different crosswalk widths, lengths, or locations of crosswalks. Pedestrian crossing safety can also be affected when there is a mismatch between the size of the guide islands and pedestrian traffic volume. In addition, the influence of pedestrians crossing the street in pairs, drivers’ physiological and psychological factors, and the turning radius of curves are all subjects worthy of in-depth study.

## 7. Conclusions

This paper analyzes the characteristics of pedestrian crossing behavior and the severity of pedestrian–vehicle conflict from “people, vehicles, roads, and environment” for pedestrian spatial violations in the advance right-turn lane, classifies the conflict level, and establishes a pedestrian–vehicle conflict analysis model based on the multivariate ordered logistic model. The main conclusions are as follows:

(1) There are significant differences in pedestrian behavior under different crossing paths. The overall pedestrian crossing speed showed a downward trend. Among them, the average speed of pedestrian crossing in path type V was the highest, which was approximately 10% higher than those of other types. When pedestrians do not use crosswalks at all, the probability of conflict with motor vehicles is the highest.
(2) In the factor analysis of the severity of the pedestrian–vehicle conflict, it was concluded that vehicle speed (4.495) and vehicle arrival rate (0.451) were positively correlated with the severity of the class conflict. Excessive speed leads to shorter reaction times for traffic participants, and greatly increases the severity of conflicts, so measures to control the speed of vehicles pulling into the right turn lane are key to ensuring pedestrian safety. The elderly (>60) are the most exposed to serious conflicts out of all the age groups. Traffic management should enhance safety education for older adults and emphasize the dangers of spatial violations. Since conflict severity is significantly higher in areas with short crosswalk lengths, warning signs should be installed in relevant areas to limit pedestrian violations.
(3) Regression analysis results show that spatial violations lead to an increase in the severity of pedestrian–vehicle conflicts, especially the irregular use of crosswalks to cross the street on the side close to the direction of traffic flow, which greatly increases the risk of crossing the street. The article obtains factors that significantly affect the severity of pedestrian–vehicle conflicts, which can be used as a theoretical basis for the implementation of regional traffic safety measures. It can also provide a reference for the establishment of pedestrian safety facilities. For example, the installation of guardrails on one side of the sidewalk to limit the starting position of pedestrians crossing the street and thus reduce space violations, etc.

## Figures and Tables

**Table 1:** Investigation site information.

Investigation Site	Crosswalk Length (m)	Crosswalk Width (m)	Surroundings	Number of Lanes
1	7.5	5	Subway stations, shopping malls	2
2	6	5	Subway stations, shopping malls	1
3	6.5	5	Subway stations, scenic spots	1
4	6.5	5	Subway stations, scenic spots	1

**Table 2:** Traffic survey data and assigned values.

Survey Variable	Description and Assignment
Gender	0: Male; 1: Female
Age	1: <10; 2: 10–20; 3: 20–40; 4: 40–60; 5: >60
Technological device	0: No device; 1: Listen to music; 2: Call; 3: Look down at the phone
Pedestrian crossing path	1: I; 2: II; 3: III; 4: IV; 5: V
Conflict with vehicles	0: No; 1: Yes
Pedestrian crossing speed	Calculation of path coordinates
Vehicle speed	Calculation of path coordinates
Vehicle arrival rates	Number of vehicles arriving per unit time (veh/min)
Pedestrian arrival rates	Number of arrivals per unit time (person/min)

**Table 3:** Cluster analysis results.

Conflict Severity Level	Potential Conflicts	Minor Conflict	Serious Conflict
Clustering Center (TTC, PET, DST)	(4.26, 5.71, 1.81)	(2.57, 3.02, 3.39)	(1.46, 1.72, 5.38)
Sample size	11	166	142

**Table 4:** Regression analysis results.

Variable	B	Standard Error	Wald	Degree of Freedom	Significance	OR
Conflict severity (1)	28.848	12.606	5.237	1	0.022	_
Conflict severity (2)	34.176	12.735	7.202	1	0.007	_
Vehicle speed	4.945	1.665	8.826	1	0.003	140.528
Vehicle arrival rate	0.451	0.205	4.844	1	0.028	1.569
Pedestrian arrival rate	0.657	2.571	3.454	1	0.154	1.214
Gender (0)	−0.001	0.291	0.000	1	0.997	0.999
Gender (1)	0 ^a^					1
Age (1)	−0.664	1.038	0.409	1	0.522	0.515
Age (2)	−4.069	0.892	20.785	1	0.000	0.017
Age (3)	−1.367	0.741	3.401	1	0.065	0.255
Age (4)	−1.666	0.800	4.330	1	0.037	0.189
Age (5)	0 ^a^					1
Technological device(0)	−0.322	0.663	0.236	1	0.627	0.725
Technological device(1)	0.110	1.242	0.008	1	0.929	1.116
Technological device(2)	1.127	1.350	0.697	1	0.404	3.087
Technological device(3)	0 ^a^					1
Path type (1)	1.432	0.578	6.127	1	0.013	4.185
Path type (2)	0.636	0.518	1.508	1	0.219	1.888
Path type (3)	−2.527	0.478	28.014	1	0.000	0.080
Path type (4)	−1.468	0.610	5.795	1	0.016	0.230
Path type (5)	0 ^a^					1
Number of lanes (1)	3.940	1.562	6.361	1	0.012	51.396
Number of lanes (2)	0 ^a^			0		1
Model fitting information	$\chi^{2}(15)=170.989\;\mathrm{LL}=188.626\;\mathrm{Pseudo}\;R2=0.328\;P<0.001$					

0 ^a^ for the reference variable.

## Data Availability

The original datasets in the study are available from the corresponding author on reasonable request.
